# A Statistical Study of the Correlation between Geomagnetic Storms and M ≥ 7.0 Global Earthquakes during 1957–2020

**DOI:** 10.3390/e22111270

**Published:** 2020-11-09

**Authors:** Hongyan Chen, Rui Wang, Miao Miao, Xiaocan Liu, Yonghui Ma, Katsumi Hattori, Peng Han

**Affiliations:** 1 Department of Earth and Space Sciences, Southern University of Science and Technology, Shenzhen 518055, China; 11930858@mail.sustech.edu.cn (H.C.); 11930870@mail.sustech.edu.cn (R.W.); miaom@sustech.edu.cn (M.M.); 2 The Institute of Geophysics China Earthquake Administration, Beijing 100081, China; liuxc@cea-igp.ac.cn; 3 School of Science, Harbin Institute of Technology, Shenzhen 518055, China; yhma@hit.edu.cn; 4 Graduate School of Science, Chiba University, Chiba 263-8522, Japan; khattori@faculty.chiba-u.jp; 5 Center for Environmental Remote Sensing, Chiba University, Chiba 263-8522, Japan

**Keywords:** statistical study, geomagnetic storm, earthquakes, superposed epoch analysis

## Abstract

In order to find out whether the geomagnetic storms and large-mega earthquakes are correlated or not, statistical studies based on Superposed Epoch Analysis (SEA), significance analysis, and Z test have been applied to the Dst index data and M ≥ 7.0 global earthquakes during 1957–2020. The results indicate that before M ≥ 7.0 global earthquakes, there are clearly higher probabilities of geomagnetic storms than after them. Geomagnetic storms are more likely to be related with shallow earthquakes rather than deep ones. Further statistical investigations of the results based on cumulative storm hours show consistency with those based on storm days, suggesting that the high probability of geomagnetic storms prior to large-mega earthquakes is significant and robust. Some possible mechanisms such as a reverse piezoelectric effect and/or electroosmotic flow are discussed to explain the statistical correlation. The result might open new perspectives in the complex process of earthquakes and the Lithosphere-Atmosphere-Ionosphere (LAI) coupling.

## 1. Introduction

The electromagnetic anomaly prior to large earthquakes has been intensively investigated during the past decades [1,2,3,4,5,6,7,8,9,10,11,12,13]. Most studies focus on the seismo-electromagnetic phenomena generated by underground activities before earthquakes or the perturbations in the atmosphere and the ionosphere, especially the ultra-low-frequency electromagnetic phenomena [14,15,16] and GPS total electron content changes [17,18,19]. In addition, some researchers studied the possible links among earthquakes, solar activity, and geomagnetic storms, and they revealed the seismicity of the earth may be related to the solar activity [20,21,22,23,24,25,26,27,28,29]. For instance, the earthquakes tend to occur in greater numbers during the sunspot maximum and the descending phase of the solar cycle [23,25]. An increase in the Kp Index before large earthquakes is also reported [30]. However, there are still active debates on the linkage between the solar-terrestrial disturbance and earthquakes. Some studies suggest insignificant solar-terrestrial triggering of earthquakes and conclude that geomagnetic storms do not trigger earthquakes [31,32].

To validate the existence of the correlation between the solar activity and the earthquakes, particularly the geomagnetic storms preceding earthquakes, we apply statistical investigation to the data of the geomagnetic storm Dst index and M ≥ 7.0 global earthquakes during 1957–2020. A stochastic test based on Superposed Epoch Analysis (SEA) is employed to test whether there is a significant increase of geomagnetic storms before large earthquakes worldwide.

## 2. Data and Methods

### 2.1. Dst Index and Earthquake Data

Dst index data reflect the equatorial horizontal component disturbances of the geomagnetic field and they are used to measure the intensity and development of a geomagnetic storm. The data record started at 1957 with a time resolution of 1-h. In this paper, the IAGA2002 Data Exchange Format of Dst index provided by the World Data Center for Geomagnetism, Kyoto (http://wdc.kugi.kyoto-u.ac.jp/) are used. In order to clarify the level of intensity, we divide geomagnetic storms into weak storms and above (Dst ≤ −30 nT), moderate storms and above (Dst ≤ −50 nT), intense storms and above (Dst ≤ −100 nT), according to the storm intensity categories of Gonzalaez et al. in 1994 [33]. Table 1 shows the proportion of the total geomagnetic storm days to the total days from January 1957 to June 2020 for different storm intensity categories.

**Figure 1:**
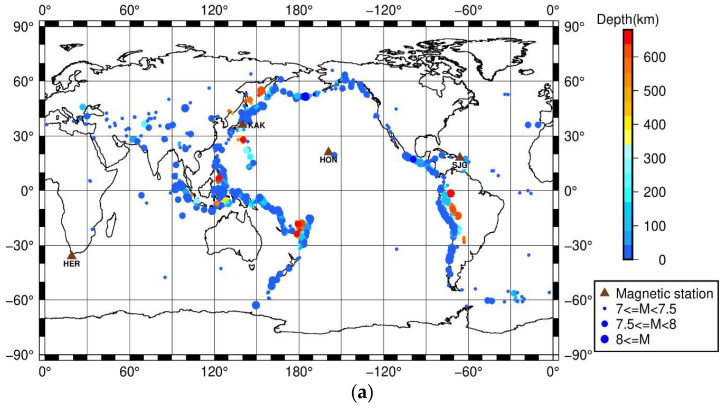

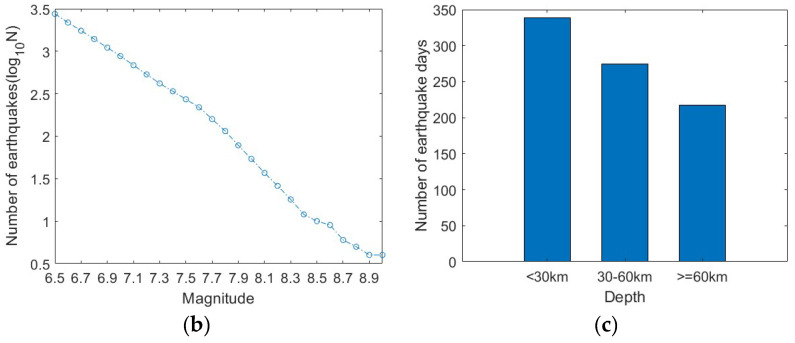
(**a**) Spatial distribution of each M ≥ 7.0 earthquake during January 1957 to June 2020; (**b**) the number of earthquakes for different magnitudes; (**c**) the number of earthquake days for different depths. The depth of the first earthquake is taken as the depth of the earthquake day if there are two or more earthquakes occurring on that day.

The earthquake data in this study include global M ≥ 7.0 earthquakes according to the United States Geological Survey (USGS) catalogue (https://earthquake.usgs.gov/earthquakes/search/). Figure 1 shows the locations of the earthquakes and the numbers of earthquakes for different magnitudes and depths. As seen in Figure 1b, earthquakes with M ≥ 7.0 are complete.

### 2.2. SEA Method

**Figure 2:**
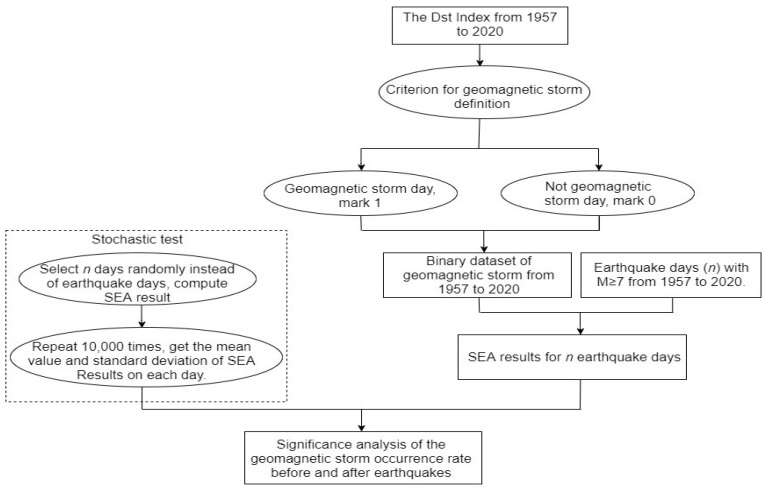
A flow chart of the SEA method.

SEA is a method to analyze the typical characteristics, periodicities, and consequences of a special event, which can be used to extract information from the time series of the finite scale containing the noise [34,35], and has been introduced in the studies of earthquakes [14,15,36]. In this paper, we apply the SEA method to test the geomagnetic storms before and after the earthquake days. First, we check the Dst index from 1957 to 2020 and define a storm day if the minimum value of the Dst indices on that day is less than a given criterion. If it is a storm day, we set the day as 1, otherwise as 0. According to this mean, we obtain a new binary dataset in terms of geomagnetic storm activities. Second, we take a sub-dataset from the binary data for each earthquake 30 days before and after its occurrence day. The timespan of each sub-dataset is 61 days and centered at the earthquake day. Third, we repeat the procedure for all the selected earthquake days and add up the counts for all data sets, then obtain the SEA result in terms of the geomagnetic storms. To evaluate the statistical significance, we use the Monte Carlo method to select random days whose number is the same as the number of earthquake days and then perform the same procedure described above to get the random SEA for geomagnetic storms. We repeat such random SEA tests 10,000 times to compute the mean, and the corresponding standard deviation of each day in 61 days’ time window. Figure 2 shows the detail flow chart of the SEA method. It should be noted that we only count one earthquake day if there are several earthquakes occurring on the same day. In such a case, the depth of the first earthquake is taken as the depth of the earthquake day.

### 2.3. Significance Analysis

**Figure 3:**
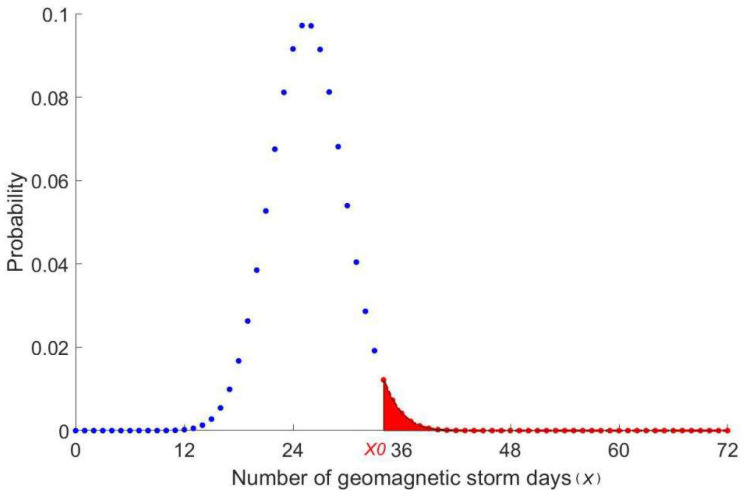
The probability distribution for obtaining different numbers of storms with *n* = 72 and *p* = 0.356. The area marked in red indicates the significance level of *x* = *x*_0_.

In addition to computing the mean and standard deviation, we also calculate the probability of the SEA results. The method has been used to analyze the lightning activities related to earthquakes [37]. In the random case, for a given day, the probabilities of obtaining *x* geomagnetic storms on that day follow a binominal distribution with parameters *n*, *p*, and *x*,

(1) $$P\left(x;n,p\right)=C_{n}^{x}p^{x}{\left(1-p\right)}^{n-x}$$

where the parameter *x* is the number of geomagnetic storms on the given day, *n* is the total number of earthquake days, and the *p* is the proportion of the total geomagnetic storm days to the total days from January 1957 to June 2020. As an example, Figure 3 shows the probability for obtaining a different number (*x*) of weak storms and above in the random case, where *p* = 35.6% and *n* = 72. The significance level of *X*_0_, i.e., the probability of obtaining *X*_0_ storms or more, is given by,

(2) $$P_{0}\left(X\geq X_{0}\right)=\overset{n}{\underset{X=X_{0}}{\sum }}P\left(x;n,p\right)$$

As a result, a small *P*_0_ value indicates that it is less likely to observe such a large value of geomagnetic storm days (*x*_0_) under this *p* in the random case.

## 3. Case Studies

**Figure 4:**
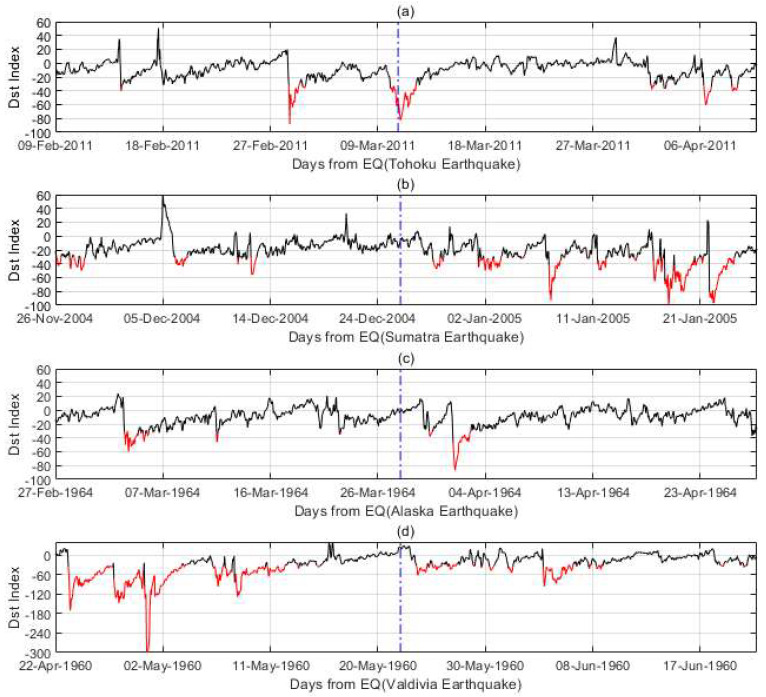
The Dst index variations in the time span of ± 30 days for four earthquakes: (**a**) Tohoku, Japan 11 March 2011 M = 9.1; (**b**) Sumatra, Indonesia 26 December 2004 M = 9.1; (**c**) Alaska, America 28 March 1964 M = 9.2; (**d**) Valdivia, Chile 22 May 1960 M = 9.5.

We start by looking at some typical cases. Figure 4 shows the Dst index data of 30 days before and after the earthquake occurrence for four mega earthquakes. The time series in red show the geomagnetic storm time when the Dst index is lower than −30 nT, and the vertical blue line centers the earthquake day. The time series in these four earthquakes show some geomagnetic activities in the time window, not only before the earthquakes, but also after the earthquakes. Figure 4d even shows a super-storm about 20 days before the 22 May 1960 Valdivia, Chile, M9.5 earthquake, which is the most powerful earthquake ever recorded. Observing geomagnetic storms in the four cases is not enough, and we cannot associate earthquakes with geomagnetic storms unless rigorous statistical testing is applied.

## 4. Statistical Analysis

**Figure 5:**
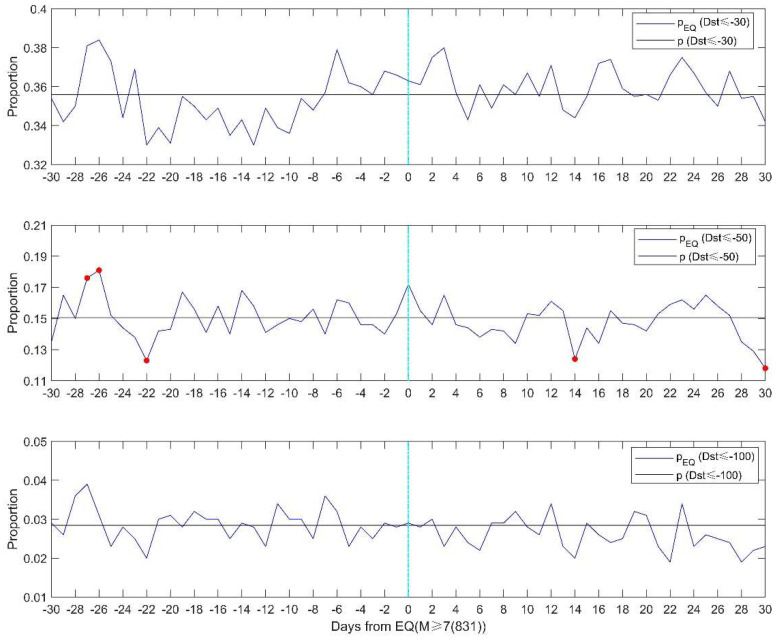
The proportion of the number of geomagnetic storm days to the total number of days (black lines) and the proportion of storm days to total earthquake days for the 831 M ≥ 7.0 earthquake days (blue lines). From top to bottom, the thresholds for storm definition are Dst ≤ −30 nT, Dst ≤ −50 nT, and Dst ≤ −100 nT, respectively. The red dot denotes 0.05 significance level of the Z test.

We then investigate the proportion of storm days to the total days (January 1957 to June 2020). The criteria for geomagnetic storm definition are listed in Table 1. Figure 5 shows the proportion of geomagnetic storm days for 831 M ≥ 7.0 earthquake days. The time window is 30 days before and after the earthquake days. We choose the 30 days is because there is one M7.0 earthquake about every 28 days from 1957 to 2020. The percentage $p_{EQ}$ is given by $\frac{N_{GS}}{N_{EQ}}$, $N_{GS}$ is the number of days with geomagnetic storms. $N_{EQ}$ is the number of M ≥ 7.0 earthquakes days. The proportion of geomagnetic storms relative to earthquakes $p_{EQ}$ is close to the proportion *p*, despite a large deviation on certain days. We apply a Z test to evaluate the statistical significance,

(3) $$z=\frac{p_{EQ}-p}{\sqrt{p\left(1-p\right)/n}}$$

where n is the number of earthquake days. The method was used to analyze the seismo-ionospheric precursors in the GPS total electron content [38]. If z > 1.96, we claim, at a significant level of 0.05, that $p_{EQ}$ > p. Some significantly high values can be seen 27 days prior to the earthquakes for Dst ≤ −50 nT.

**Figure 6:**
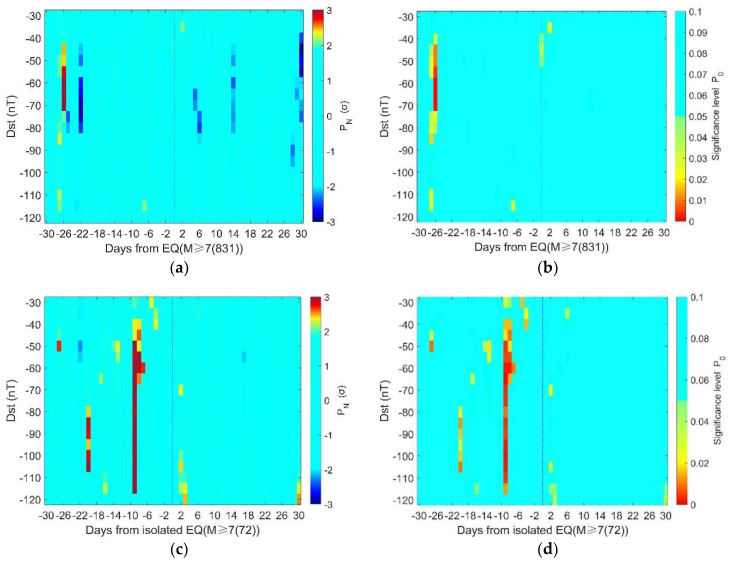
(**a**) Normalized probabilistic intensity ($P_{N}$) for the 831 M ≥ 7.0 earthquake days; (**b**) the significance level of geomagnetic storms in relation to the 831 M ≥ 7.0 earthquake days; (**c**) the normalized probabilistic intensity ($P_{N}$) for the 72 M ≥ 7.0 ‘isolated’ earthquake days; (**d**) the significance level of geomagnetic storms in relation to the 72 M ≥ 7.0 ‘isolated’ earthquake days. Note that in panels (**a**,**c**), *P_N_* within ± 2σ is presented in the same color. In panels (**b**,**d**), the color bar only extends to 0.1, and those above 0.1 are presented in the same color.

To clarify the correlation between geomagnetic storms and large-mega earthquake events, we conduct statistical analysis systematically by using different thresholds for geomagnetic storm definition. Figure 6a, b shows the SEA results for the all 831 earthquake days with M ≥ 7.0 during 1957–2020. The thresholds change from Dst ≤ −30 nT to Dst ≤ −120 nT with a step of 5 nT. Figure 6a presents the distribution of geomagnetic anomalies relative to earthquakes in terms of normalized probabilistic intensity ($P_{N}$), which is obtained by:

(4) $$P_{N}=\frac{N_{GS}-N_{RM}}{\sigma }$$

where $N_{GS}$ is the number of days with geomagnetic storms, $N_{RM}$ and *σ* are the mean and the corresponding standard deviation computed by the 10,000 random SEA. Figure 6b shows the significance level of geomagnetic storms in relation to earthquakes using the method in Section 2.3. It could be found that in both figures there are higher probabilities of geomagnetic storms about 27 days before the earthquakes.

As there might be more than one earthquake in the time interval of 61 days, the statistical results may be affected by the temporal distribution of earthquakes, particularly when there is a large number of aftershocks with M ≥ 7.0. To rule out such influence, we take statistical investigation of the ‘isolated’ earthquakes for which there is no other M ≥ 7.0 earthquake 30 days before and after. The results are given in Figure 6c,d. There are clearly higher probabilities of geomagnetic storms prior to the earthquakes than after them. The most prominent increase of geomagnetic storms appears on 9 days before ‘isolated’ M ≥ 7.0 earthquakes.

**Figure 7:**
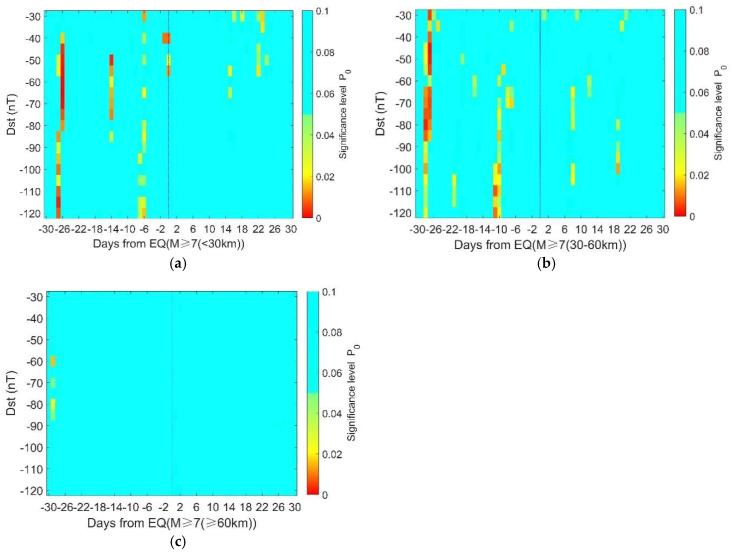
The significance level of geomagnetic storms in relation to earthquakes with different depths. (**a**) Depth <30 km, 339 earthquake days; (**b**) depth 30–60 km, 275 earthquake days; (**c**) depth ≥ 60 km, 217 earthquake days. Note that the color bar only extends to 0.1 and those above 0.1 are presented in the same color.

Figure 7 shows the significance level of geomagnetic storms in relation to earthquakes with different depths. Figure 7a,b both show more areas with a higher significance level before earthquakes than after it, suggesting that there are more geomagnetic storms prior to large-mega earthquakes. The common period for observing more geomagnetic storms is around 27 days before the earthquakes. However, this phenomenon is not clear for deeper cases (≥60 km) as shown in Figure 7c, implying that geomagnetic storms are more likely to be related with shallow earthquakes rather than deep ones.

## 5. Discussion

### 5.1. The Statistical Results on Cumulative Hours of Geomagnetic Storms

**Figure 8:**
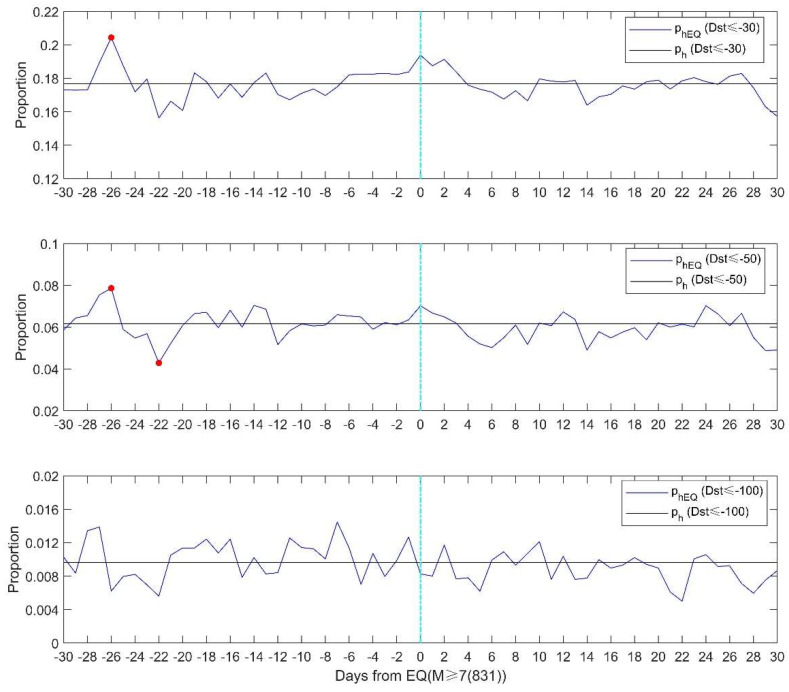
The proportion of the number of geomagnetic storms hours to the total number of hours (black lines) and the proportion of storm hours to total hours on a given day for the 831 M ≥ 7.0 earthquake days (blue lines). From top to bottom, the thresholds for storm definition are Dst ≤ −30 nT, Dst ≤ −50 nT and Dst ≤ −100 nT, respectively. The red dot denotes a 0.05 significance level of the Z test.

**Figure 9:**
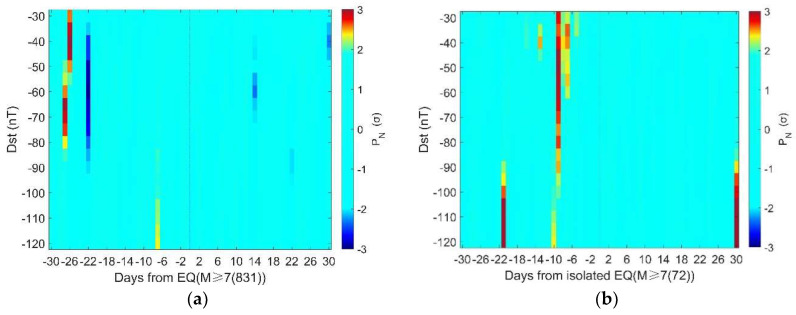
The SEA result using geomagnetic storm cumulative hours: (**a**) The result from a Superposed Epoch Analysis (SEA) showing the Dst-Time spectrogram for 30 days before and after the occurrence day of 831 M ≥ 7.0 earthquake days of normalized probabilistic intensity constructed from random days. (**b**) The result from a Superposed Epoch Analysis (SEA) showing the Dst-Time spectrogram for 30 days before and after the occurrence day of 72 ‘isolated’ M ≥ 7.0 earthquake days of normalized probabilistic intensity constructed from random days. Note that *P_N_* within ± 2σ is presented in the same color.

In Section 4, if there is more than one hour with the Dst index satisfying the criterion, we count the day as a geomagnetic storm day. This may not reflect the accurate storm activity because the geomagnetic storm has a certain duration, including the main phase and recovery phase. Further analysis to check the statistical result is applied by calculating the cumulative hours of geomagnetic storms each day. Figure 8 shows the Z test based on the cumulative hours of geomagnetic storms. $p_{h}$ is the proportion of the total geomagnetic storm hours to the total number of hours from January 1957 to June 2020. $p_{hEQ}$ is the proportion of storm hours to total hours on a given day for the 831 M ≥ 7.0 earthquake days. The Z test is applied according to Formula (3). Figure 9 shows the SEA results of the cumulative hours of geomagnetic storms for 30 days before and after earthquake occurrence.

The Z test in Figure 8 shows that there are higher proportions of weak and moderate storms 26 days prior to earthquakes. Comparing Figure 9a with Figure 6a, we can find that the probability of geomagnetic storms clearly increases around 26–27 days before earthquakes. As for the ‘isolated’ earthquakes, both Figure 9b and Figure 6b show similar results. The number of geomagnetic storms is significantly high on 9 and 21 days before ‘isolated’ earthquakes. These consistencies suggest that the high probability of geomagnetic storms prior to large-mega earthquakes is significant and robust.

### 5.2. The Dependence on Depth

**Figure 10:**
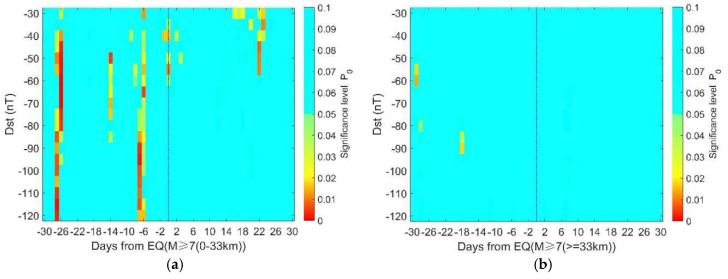
The significance level of geomagnetic storms in relation to earthquakes with different depths. (**a**) The result of 30 days before and after the occurrence day for 378 M ≥ 7.0 earthquake days and depth at < 33 km. (**b**) The result of 30 days before and after occurrence day for 453 M ≥ 7.0 earthquake days and depth at ≥ 33 km. Note that the color bar only extends to 0.1 and those above 0.1 are presented in the same color.

The statistical results of M≥7.0 earthquakes with different depths are shown in Section 4, and we can see the results of shallow earthquakes are apparently different from those of the deep ones. In this part, we divide the M ≥ 7.0 earthquakes catalog into two groups, i.e., upper or under 33 km. We choose 33 km as the boundary because there are many earthquakes occurring at this depth, and it could give the minimum difference between the numbers of earthquakes in the two groups. Figure 10 shows that there are clearly more geomagnetic storm days prior to earthquakes with depth < 33 km, particularly on 27 and 7 days before. In contrast, there are almost no significant periods in the result of the earthquakes with depths of ≥ 33 km. These results imply that geomagnetic results are more likely to be related with shallow earthquakes.

### 5.3. Interpretation

In this study, we find that there are significantly high probabilities of geomagnetic storms prior to global M ≥ 7.0 earthquakes. The significance persists when changing the thresholds of storm definition from −45 nT to −70 nT for the overall 831 earthquake days, as shown in Figure 6a,b. The statistical results are even more stable for the 72 ‘isolated’ earthquake days. The significance holds on 9 days before isolated earthquakes when the thresholds change from −40 nT to −115 nT, as shown in Figure 6c,d. These suggest that the statistical significance is not so sensitive to the criterion chosen for storm definition. On the other hand, there is clear depth dependence in the statistical results. These phenomena imply that the correlation between geomagnetic storms and large-mega earthquakes is not likely to be a statistical coincidence.

To evaluate the significance level, we randomly select earthquake days and compute the statistical significance. However, the randomly chosen events may not exhibit the same clustering feature as the actual earthquakes. To eliminate the influence of clustering in earthquake samples, one could remove aftershocks using some declustering approaches. In this study, as an attempt, we proposed another method to minimize the influence of the cluster effect by introducing the “isolated earthquakes”. The results suggest that there are clearly more magnetic storms before M ≥ 7.0 isolated earthquakes, though the significance period becomes different from those using all earthquakes.

In Figure 6b, the proportion of significance area (P_0_ ≤ 0.05) to the total is about 1.9%. The proportion increases to 4.23% in Figure 6d. This implies that the correlation between geomagnetic storms and ‘isolated’ earthquakes is more evident than that between geomagnetic storms and overall earthquakes. In other words, the clustering in the earthquake samples may obscure the correlation. The proportion of the significance area is 7.1% in Figure 10a, while it is less than 0.5% in Figure 10b, suggesting a clear depth dependence of the correlation between storms and earthquakes.

In a completely random case, we could observe or generate synthetic earthquake time series and 5% of them could give significant results. So, the question is: “whether the actual earthquake time series is generated by chance or induced, even partially, by geomagnetic storms”. In either case, we cannot deny the correlation between the two actual time series. But the correlation does not suggest causality. We could not infer a causal link between geomagnetic storms and earthquakes, unless (1) the correlation always exists and (2) the physical mechanism behind is clear and proved. For example, it has been claimed that oil price was correlated with earthquakes in China. This correlation lacks a known causal basis, and by retrospectively focusing attention on its presence, one might be seduced by “inspection bias” [39]. To avoid this, the correlation should be shown to be both persistent and detectable in a second dataset that was not used in the original identification of the correlation [31]. In Figure 7a, we find the significance on 27 days before earthquake for depth < 30 km, and the significant value is persistent and detectable before the earthquake for depths of 30–60 km in Figure 7b. Besides, the significant values appear only before earthquakes in Figure 9b when the cluster effect is eliminated. If the result is occasional, we may also find significant value in the post-earthquake period. Thus, there might be causality between the geomagnetic storms and earthquakes. The possible physical links are discussed below.

So far, researchers have proposed several mechanisms about Lithosphere-Atmosphere-Ionosphere (LAI) coupling during the preparation process of earthquakes [40,41]. For example, Vito Marchitelli et al. analyzed the correlation between 20-year proton density and large earthquakes worldwide and argued that such a relationship could be explained with a reverse piezoelectric effect [28]. According to previous studies [42], large electric currents could generate stress changes in rocks. As the Earth is conductive, large currents could be expected during geomagnetic storms. Consequently, a perturbation in stress filed on the fault might happen. Mizutani et al. examined the electrokinetic phenomena associated with earthquakes and argued that the principle of electrokinetic phenomena may be used for earthquake control by controlling the water flow with an artificial electric potential field [43]. The geomagnetic storms can provide such an electric potential field via an induction effect and may lead to ground fluid flow. This process is the electroosmosis [44]. The fluid migration could change the pore-pressure and fault strength which are essential parameters for rupture. Therefore, the electroosmotic flow driven by large induced currents during geomagnetic storms might be another possible mechanism. Urata et al. reviewed several studies and claimed that magnetic field variations created electric “telluric” currents and generate additional mechanical forces in seismic rupture zones [30]. These explanations take the geomagnetic storms as a triggering effect to some extent. Other explanations take the geomagnetic disturbance as precursors induced by underground stress evolution during the preparation process of earthquakes. When the fault system goes into the critical stage, electric currents may appear in lithosphere [45,46,47,48]. These currents may change the motion of charged particles in the atmosphere and ionosphere [49] and cause perturbations in ionosphere and magnetosphere [50,51]. As for our cases, the geomagnetic storms are quite strong perturbations and we tend to believe it is not likely to be induced by possible ground currents. The reverse piezoelectric effect and/or electroosmotic flow are more reasonable explanations for the statistical correlation at present.

## 6. Conclusions

In this study, we systematically investigated the correlation between geomagnetic storms and M ≥ 7.0 global earthquakes during 1957–2020. The results give the statistically significant evidence that more geomagnetic storms occur before global M ≥ 7.0 earthquakes. Further statistical investigations of the results using cumulative storm hours show consistency with those using storm days, suggesting that the high probability of geomagnetic storms prior to large-mega earthquakes is significant and robust. Moreover, we check the dependence on the depth and find that the increase of geomagnetic storms becomes inconspicuous before deep earthquakes, implying that geomagnetic storms are more likely to be related with shallow earthquakes rather than deep ones. Although there is no quantitative model to explain such a phenomenon, we believe that some physical mechanism such as the reverse piezoelectric effect and/or electroosmotic flow are promising candidates to interpretate the statistical results. The mechanisms of geomagnetic storm observation before the earthquakes are worth studying in the future.

## Figures and Tables

**Table 1:** The proportion of the total geomagnetic storm days to the total days.

Category	Dst(nT)	Percentage (*p*)
Weak storm and above	Dst index ≤ −30	35.6%
Moderate storm and above	Dst index ≤ −50	15.1%
Intense storm and above	Dst index ≤ −100	2.84%
